# Impact of anxiety profiles on cognitive performance in BALB/c and 129P2 mice

**DOI:** 10.3758/s13415-012-0109-7

**Published:** 2012-07-04

**Authors:** Amber R. Salomons, Saskia S. Arndt, Frauke Ohl

**Affiliations:** 1 Division of Animal Welfare & Laboratory Animal Science, Department of Animals in Science and Society, Faculty of Veterinary Medicine, Utrecht University, Yalelaan 2, P.O. Box 80.166, 3508 TD Utrecht, The Netherlands; 2 Rudolf Magnus Institute of Neuroscience, Utrecht, The Netherlands

**Keywords:** Cognition, Anxiety, Behaviour, 129P2 mice, BALB/c mice, Habituation

## Abstract

It has been suggested over the decades that dysfunctional anxiety may be caused by distinct alterations in cognitive processing. To learn more about the relation between anxiety and cognitive functioning, two mouse strains that display either adaptive (BALB/c) or nonadaptive (129P2) anxiety, as reflected by their ability to habituate when repeatedly exposed to a novel environment, were tested for their cognitive performance in the modified hole board (mHB) task. In general, both strains showed successful acquisition of the task. The initially more anxious BALB/c mice revealed rapid habituation to the test setup, followed by decreased long-term and short-term memory errors across the experimental period and fast relearning after reversal of the task. By contrast, the nonadaptive 129P2 mice made more short-term memory errors and performed worse than the BALB/c animals after reversal of the test. The results confirm the proposed interaction of anxiety and cognition: In BALB/c mice, adaptive characteristics of anxiety were paralleled by more successful cognitive performance, while in 129P2 mice nonadaptive anxiety-related behaviour was accompanied by a higher level of short-term memory errors and less cognitive flexibility. Moreover, these results support our hypothesis that the nonadaptive anxiety phenotype in 129P2 mice may be the result of impaired cognitive control of emotional processes, resulting in impaired behavioural flexibility, for example in response to novelty.

Anxiety is a fundamental emotional response to real or potentially threatening stimuli that is accompanied by behavioural, neurological, and physiological responses (Livesey, 1986). Emerging evidence has shown that anxiety and cognition are closely associated and interacting processes (Beuzen & Belzung, 1995; McNaughton, 1997). For example, cognitive impairments in humans suffering from anxiety disorders have been described (Castaneda, Tuuio-Henriksson, Marttunen, Suvisaari, & Lonnqvist, 2008; Ferreri, Lapp, & Peretti, 2011), and it has been suggested that pathological variants of anxiety may be based on a primarily cognitive dysfunction in that inadequate anxiety responses may arise when there is a mismatch between the information perceived and the information already stored. Such a mismatch may then result in impaired integration at a higher cognitive level and could, as a consequence, eventually lead to inappropriate anxiety responses (Gray, 1990; Hindmarch, 1998; McNaughton, 1997). Furthermore, there is ample evidence that, for example, emotional arousal modulates memory formation, and thus cognitive processing of such emotional events (Cahill & McGaugh, 1998; McGaugh, Cahill, & Roozendaal, 1996). At the central nervous level, the brain structures involved in anxiety in animals, such as the hippocampus and amygdala, have been implicated in both memory processes and anxiety. In addition, compounds that reduce anxiety have been found to cause amnesia in both animals and humans (Kostowski, Płaźnik, & Stefański, 1989; LeDoux, 1992). However, contrary results have been found, in that increased anxiety has been associated with either cognitive deficits (e.g., Silva & Frussa-Filho, 2000) or an enhancement in emotional memory (e.g., Adreatini, Wolfman, Viola, Medina, Da Cunha & Ribeiro, 1999), and it remains unclear to what extent cognitive (dys)function may precede or, in contrast, result from a specific type of anxiety disorder (Garner, Mohler, Stein, Mueggler, & Baldwin, 2009). Especially, the relationship between *pathological* anxiety and cognitive functioning remains to be investigated.

Animals that are characterised by different levels of innate anxiety are used as a model to study the interaction between anxiety and cognitive functioning. For example, in rats bred for high and low anxiety-related behaviour, different levels of anxiety have been found to be associated with specific cognitive performance in a visuospatial task (Ohl, Roedel, Storch, Holsboer, & Landgraf, 2002). Using the same test setup, the initially highly anxious DBA mouse strain revealed a different exploratory strategy in comparison with the low-anxiety C57BL/6 strain, resulting in fewer memory errors over time (Ohl, Roedel, Binder, & Holsboer, 2003). These and other results (Beuzen & Belzung, 1995; Hindmarch, 1998; McNaughton, 1997) have confirmed a close correlation between anxiety and cognition. Interestingly, the highly anxious rats and mice in these studies all showed efficient habituation to the test setup over time, indicating perhaps that these animals display adaptive, nonpathological anxiety (Ohl et al., 2002; Salomons, van Luijk, Reinders, Kirchhoff, Arndt & Ohl, 2010).

In recent experiments, we have shown that two parental mouse strains of the 129 mouse family (129P2 and 129P3) are characterised by impaired habituation in response to repeated exposure to a mildly aversive environment, while by contrast the initially highly anxious BALB/c mice showed rapid adaptation to a similar environment (Salomons et al., 2010). Interestingly, the nonadaptive anxiety profile of 129P3 mice, indicated by a lack of habituation to the test setup, was accompanied by lower activation of the prefrontal cortex when compared to BALB/c mice, while the two strains did not differ in their extents of amygdala activation or in plasma corticosterone levels (Salomons et al., 2010). Notably, we found that 129P3 mice did not perform differently from BALB/c individuals in an object recognition task, suggesting that their habituation behaviour was unlikely to be the result of a general cognitive impairment. However, these mouse strains have not yet been compared for more complex cognitive functions, such as long-term/short-term memory and cognitive flexibility.

Therefore, to elucidate the relation between anxiety-related habituation and cognitive processing, we determined the cognitive performance of a member of the 129 mouse family, the 129P2 strain, and compared it to the performance of the BALB/c strain. For this study, we used a modified hole board (mHB) task, which is a visuospatial task that allows for the simultaneous evaluation of different cognitive processes and other behavioural dimensions, such as avoidance behaviour, risk assessment, exploration, and locomotion over time (Ohl et al., 2003; Ohl et al., 2002; van der Kooij, Ohl, Arndt, Kavelaars, van Bel, Heijnen, 2010).

## Method

### Ethical note

The experimental protocols were reviewed and approved by the Animal Experiments Committee of the Academic Biomedical Centre Utrecht, The Netherlands. The Animal Experiments Committee based its decision on the Dutch implementation of the EC Directive 86/609/EEC (Directive for the Protection of Vertebrate Animals Used for Experimental and Other Scientific Purposes). Furthermore, all animal experiments followed the national Code on Laboratory Animal Care and Welfare and were performed with reference to the *Guidelines for the Care and Use of Mammals in Neuroscience and Behavioural Research* (National Research Council, 2003).

### Animals and housing

Naïve male 129P2/OlaHsd (129P2, *n* = 10) and male BALB/cOlaHsd (BALB/c, *n* = 10) mice were obtained from Harlan (U.K.) and arrived at the age of 7–8 weeks at the animal facilities of Utrecht University. The mice were housed individually in Eurostandard Type II cages with bedding material (aspen chips), a tissue and shelter for cage enrichment, and tap water and food ad libitum. The mice were kept in the experimental room under a reversed dark–light cycle (lights on between 18.00 h and 6.00 h) for 17 days for acclimatisation. During this period, all of the animals were handled three times a week between 9.00 and 11.00 h by the experimenter. All behavioural testing took place in the animal’s housing room, and the equipment (including the behavioural test setup) was installed before the animals arrived. Relative humidity was kept at a constant level of approximately 50 %, room temperature was sustained at 22º ± 2 °C, and the ventilation rate was 15–20 air changes per hour.

### Modified hole board task

**Fig. 1:**
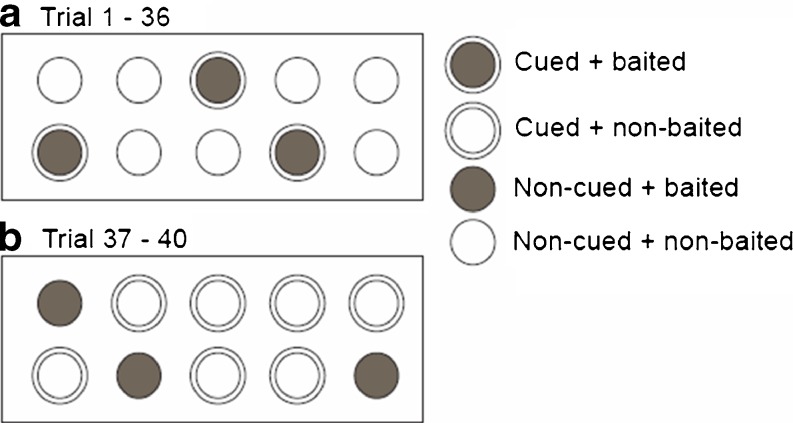
Schematic representation of the modified hole board task. The animals were tested for four trials per day over five consecutive days. After 2 days’ rest, the testing continued for another 5 days (four trials per day). Until Testing Day 9 (Phase 1, Trials 1–36), the hole board consisted of three cued and baited cylinders, with seven cylinders noncued and nonbaited (A). On Day 10 (Phase 2, Trials 37–40; *reversal*), a new set of baited cylinders was presented. This set was noncued, while the seven nonbaited cylinders were cued (B)

Behavioural testing was done with the cognitive version of the mHB, according to a protocol that we have described previously (see Ohl et al., 2003; Ohl et al., 2002; van der Kooij et al., 2010). The mHB setup was made of an opaque grey PVC box (50 × 50 × 50 cm) containing a hole board (35 × 20 cm) placed in the middle of the box (Fig. 1), in which 10 cylinders were positioned in two rows. The cylinders were flavoured with vanilla (vanilla flavour dissolved in water [0.02 %]) as mice are attracted by this flavour. All 10 cylinders were baited with a small piece of almond fixed underneath a metal grid, which could not be removed by the animals. Three of the 10 cylinders were marked with a white ring, and the piece of almond (0.05 g) was placed on top of the metal grid, so that it could be retrieved by the animals (baited cylinders). For 3 days prior to behavioural testing, each animal received a piece of almond in its home cage to habituate to the food. Testing took place between 9.00 and 13.00 h, during the early activity phase of the animals. One animal at a time was transferred directly from the home cage to the mHB and allowed to explore the area until all three food rewards had been retrieved or for a maximum trial duration of 5 min, whichever event occurred first. The mice were tested in close succession, but at least 5 min were allowed between testing of one mouse and the next, in order to clean and rebait the holeboard.

All trials were immediately monitored and scored by a trained observer, using the software program Observer 5.0 (Noldus Information Technology, Wageningen, The Netherlands). After testing, the animals were transferred back to their home cages, and the mHB was carefully cleaned with tap water and paper towels.

The first testing phase consisted of four trials per day with three cued and baited cylinders, while seven were noncued and nonbaited, over five consecutive days (Phase 1, see Fig. 1 top). The testing was interrupted for 2 days, and on the 6th day testing continued with the same setup. The reversal task was performed on Day 10: The locations of the three baited cylinders was scrambled, and these cylinders were noncued, while the seven remaining, nonbaited cylinders were cued (Phase 2, Fig. 1 bottom).

The following behavioural categories were measured: cognition, avoidance behaviour, risk assessment, locomotor activity, exploration, and arousal (Table 1). The latency to complete the trial and the latency until the first visit of a baited cylinder were measured as indicators of cognitive performance and motivation. Furthermore, visits of nonbaited cylinders were defined as wrong choices and regarded as a long-term memory error (Squire & Zola, 1996), since recollection of the previously learned information (which cylinders were baited) was necessary to avoid wrong choices. Revisits to a baited cylinder indicated a short-term memory error (Goldman-Rakic, 1996), as animals needed to learn which cylinders they had already visited. Additionally, nonvisited baited cylinders were defined as omission errors. Notably, both the numbers of long-term and short-term memory errors represented overestimates of the actual error rate as long as omission errors were performed. However, rather than correcting the short- and long-term errors for the number of omissions, we chose to present all three error types.

**Table 1:** Cognitive and behavioural parameters measured in the modified hole board task

Behavioural Category	Behavioural Parameter
Cognition	Latency to complete the task (find all three food rewards) [s]
	Latency until first visit baited cylinder [s]
	Omission errors (number of food rewards not found) [*N*]
	Revisit baited cylinders (short-term memory errors) [*N*]
	Visit nonbaited cylinders (long-term memory errors) [*N*]
	Wrong choice (revisit baited cylinder + visit nonbaited cylinder) [*N*]
Avoidance behaviour	Latency until first board entry [s]
	Total time spent on board [%]
	Total number of board entries [*N*]
Risk assessment	Total number of stretched attends [*N*]
	Latency until first stretched attend [s]
Locomotor activity	Total number of line crossings [*N*]
	Latency until first line crossing [s]
	Total time spent immobile [%]
	Latency until first immobility event [s]
Exploratory activity	Total number of rearings in the box [*N*]
	Total number of rearings on the board [*N*]
	Total number of cylinder explorations [nr]
Arousal	Total time spent self-grooming [%]
	Latency until first self-grooming event [s]
	Defecations [*N*]

### Statistical analyses

Statistical analyses were performed with SPSS (Version 16.0, SPSS Inc., IL). Continuous data (latency and relative duration) were represented as means with standard errors of the means (± *SEM*s) and were first tested for Gaussianness using the Kolmogorov–Smirnov test. Homoscedasticity was tested by Levene’s test. Data that revealed a nonparametric distribution and all of the behavioural parameters (represented as medians with interquartile ranges [IQRs]) were rank transformed (Conover & Iman, 1982). The (transformed) data were subsequently analyzed with repeated measures ANOVAs using Strain as a between-subjects factor and Trial as a within-subjects factor. Sphericity was tested with Mauchly’s test. A significant result in Mauchly’s test was corrected either by a Huynh–Feldt correction (estimates of sphericity > .75) or by a Greenhouse–Geisser correction (estimates of sphericity < .75; Girden, 1992). Post hoc analyses were done using an unpaired Student’s *t* test for continuous data and the Mann–Whitney *U* test for discrete data. For the ANOVA analyses, a probability value less than .05 was considered to be statistically significant. To minimize the risk of a Type I error due to multiple comparisons, the level of significance was corrected for the post hoc analyses using the Dunn–Sidak correction (Ludbrook, 1991).

## Results

### Cognitive performance: Phase 1

**Fig. 2:**
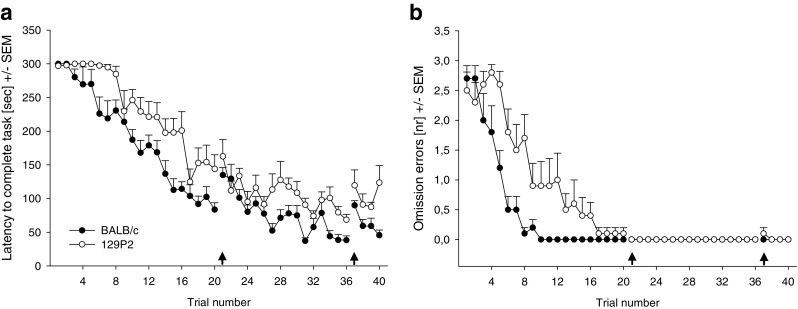
Cognitive parameters measured in BALB/c and 129P2 mice. Data are shown as averages per trial (four trials/day) ± *SEM*s. (A) Latencies to complete the task (i.e., all three food rewards found). (B) Number of omission errors (i.e., number of baited cylinders not visited). The first arrow indicates a break of 2 days; the second arrow indicates the reversal task (on Day 10)

Main effects of trial were found for latencies to complete the task [*F*(22.6, 361.6) = 59.609, *p* < .001; Fig. 2A], latencies until the first baited cylinder visit [*F*(11.2, 178.8) = 59.007, *p* < .001], and the number of omission errors [*F*(8.9, 143.8) = 42.250, *p* < .001; Fig. 2B]. Both strains showed a decrease in all of these parameters over the course of the experiment, resulting in less time needed to complete the trials. Omission errors were absent after the 20th trial, indicating that all mice had learned to find the three food rewards. Strain and interaction effects were found for these parameters as well [latency to complete the task, *F*(1, 16) = 19.317, *p* < .001; latency until the first baited cylinder visit, *F*(1, 16) = 5.860, *p* < .05; *F*(11.2, 178.8) = 2.408, *p* < .001; omission errors, *F*(1, 16) = 8.189, *p* < 0.01; *F*(8.9, 143.8) = 4.088, *p* < .001]. Post hoc testing showed that 129P2 mice in general needed more time to complete the task, which was significant for Trials 3, 5, 6, 7, 16, and 31 (*p*s < .001). In addition, 129P2 mice made more omission errors than did BALB/c mice in Trials 8 and 10–16 (*p*s < .001).

**Fig. 3:**
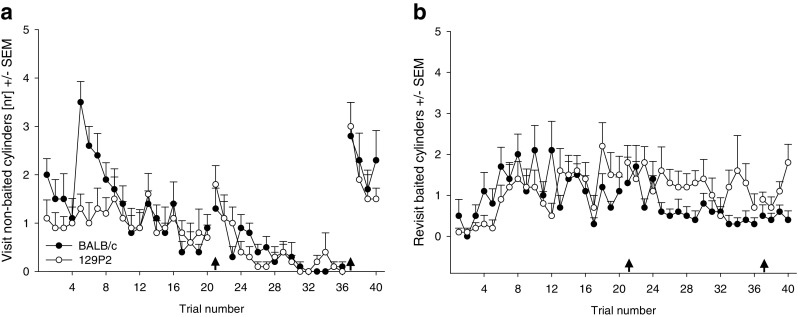
Cognitive parameters measured in BALB/c and 129P2 mice. Data are shown as averages per trial (four trials/day) ± *SEM*s. (A) Number of visits to nonbaited cylinders (as indicator for long-term memory errors). (B) Number of revisits to baited holes (as indicator for short-term memory errors). The first arrow indicates a break of 2 days; the second arrow indicates the reversal task (on Day 10)

For the number of visits to nonbaited cylinders (Fig. 3A), indicating long-term memory errors, effects of trial [*F*(33.8, 541.8) = 9.793, *p* < .001] and strain [*F*(1, 16) = 6.546, *p* < .05], as well as a Strain × Trial interaction [*F*(33.8, 541.8) = 1.477, *p* < .05], were found. While both strains showed a decrease in the number of nonbaited cylinder visits, BALB/c mice visited the nonbaited cylinders significantly more than the 129P2 mice did. Trial [*F*(35.9, 574.3) = 2.421, *p* < .001], strain [*F*(1, 16) = 8.439, *p* < .01], and interaction [*F*(35.9, 574.3) = 2.452, *p* < .01] effects were also found for the number of revisits of the baited cylinders (Fig. 3B), reflecting short-term memory errors, with 129P2 mice revealing more such errors than BALB/c animals. A combined analysis for the total number of errors—short- and long-term—revealed a trial effect [*F*(32.9, 527.1) = 4.508, *p* < .001] and a Trial × Strain interaction [*F*(32.9, 527.1) = 1.757, *p* < .01], with BALB/c making more errors overall, while both strains showed a gradual decline in the number of errors during the course of the experiment.

### Overall behaviour: Phase 1

**Fig. 4:**
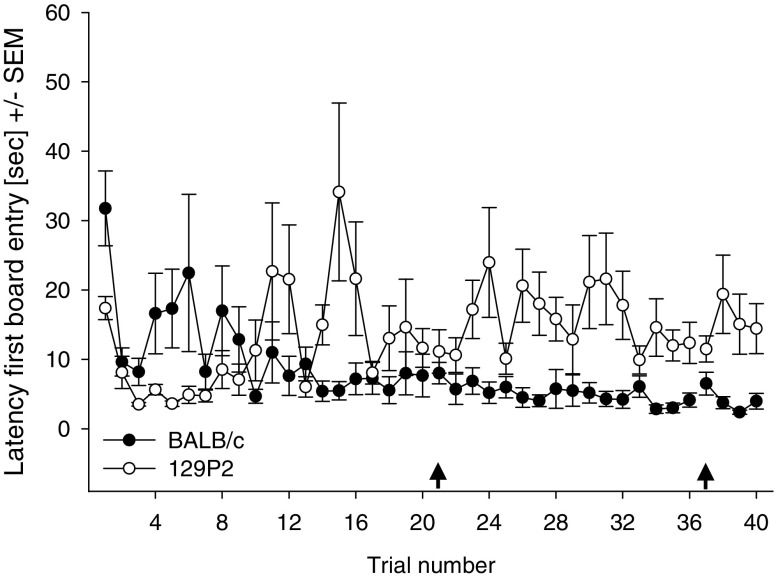
Behavioural parameters measured in BALB/c and 129P2 mice: Latencies until the first board entry, shown as averages per trial (four trials/day) ± *SEM*s, as a measure of avoidance behaviour. The first arrow indicates a break of 2 days; the second arrow indicates the reversal task (on Day 10)

A main effect of trial [*F*(12.8, 205.4) = 2.242, *p* < .05] and a Strain × Trial interaction [*F*(12.8, 205.4) = 4.305, *p* < .001] were found for latencies until the first board entry (Fig. 4): While BALB/c mice showed a decrease in avoidance behaviour over time, 129P2 mice showed a decrease and then a slight increase in avoidance behaviour. 129P2 mice showed a longer latency to enter the board when compared to BALB/c mice only in Trial 14 (*p* < .001). For the time spent on the board, main effects of trial [*F*(14.3, 228.3) = 5.156, *p* < .001] and strain [*F*(1, 16) = 27.748, *p* < .05] and a Strain × Trial interaction [*F*(14.3, 228.3) = 2.105, *p* < .01] were found. While both strains spent less time on the board with increasing trials, BALB/c mice initially spent less time on the board when compared to 129P2 mice. This difference between the strains was no longer present after the first week of testing. Risk assessment behaviour, reflected by the number of stretched attends, decreased across the experimental period [trial, *F*(5.9, 95.1) = 104.704, *p* < .001; Trial × Strain, *F*(5.9, 95.1) = 8.925, *p* < .001] and was completely absent in the second week of testing in both strains. Post hoc testing revealed that BALB/c mice made more stretched attends than the 129P2 mice only in the first trial (*p* < .001).

For the number of line crossings, significant effects of trial [*F*(14.1, 226.1) = 10.855, *p* < .001] and strain [*F*(1, 16) = 34.937, *p* < .05] and a Strain × Trial interaction [*F*(14.1, 226.1) = 2.141, *p* < .01] were observed. Both strains showed an increase in the number of line crossings from Trial 1 onwards. Post hoc testing revealed that BALB/c mice made significantly more line crossings on the 6th day of testing (Trials 21–24, *p* < .001).

Trial [*F*(21.9, 351.8) = 4.424, *p* < .001] and strain [*F*(1, 16) = 16.340, *p* < .001] effects were found for the exploration parameter “rearings in the box.” While both strains showed an increase in the number of rearings across the experimental period, BALB/c mice showed more rearings than 129P2 mice. Furthermore, significant trial [*F*(32.9, 527.5) = 30.987, *p* < .001], strain [*F*(1, 16) = 14.891, *p* < .05], and Strain × Trial interaction [*F*(32.9, 527.5) = 2.141, *p* < .01] effects were observed for cylinder explorations. Both strains show a gradual decrease in the number of cylinder explorations during the course of the experiment, but BALB/c mice overall showed more cylinder explorations than did 129P2 mice.

Strain effects were found for the arousal-related parameters “latency until the first grooming event” [*F*(1, 16) = 25.345, *p* < .001] and “time spent grooming” [*F*(1, 16) = 14.630, *p* < .001]. Overall, BALB/c mice spent more time grooming. Defecation did not differ between the strains but did generally decrease over the course of the experiment [trial: *F*(17.8, 285.1) = 10.710, *p* < .001].

### Cognitive performance: Reversal task

**Fig. 5:**
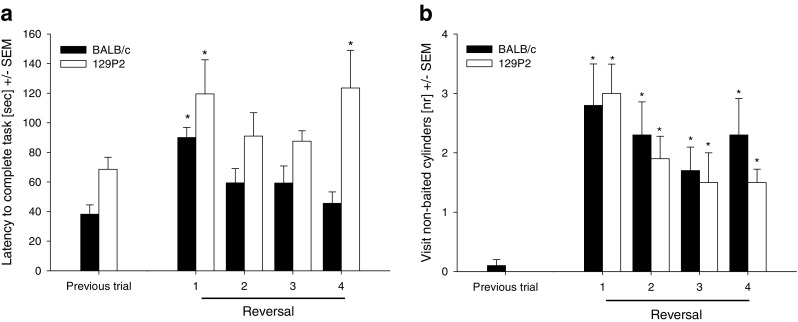
Cognitive parameters measured in BALB/c and 129P2 mice in the modified hole board during the reversal task. Data are shown as Trial 36 (previous trial) versus the reversal trials (Trials 37–40). (A) Latencies until finishing the trial (i.e., all three food rewards found). (B) Number of nonbaited cylinder visits, as an indicator for long-term memory errors. ^*^ *p* < .0085 (vs. previous trial)

Both strains showed increases in the latency to complete the task during the first reversal trial (BALB/c, *t* = −5.558, *p* < .0085; 129P2, *t* = −4.588, *p* < .0085; Fig. 5A), indicating that the mice needed more time to complete the task than on the previous trial. While the latency to complete the task steadily decreased again in the second and third reversal trials in both strains, 129P2 mice showed a significant increase in the last reversal trial (*t* = −3.508, *p* < .0085, Fig. 5A). BALB/c mice needed more time to find the first food reward, but only in the first reversal trial (*t* = −3.207, *p* < .0085). No omission errors were made in the reversal trials, indicating that all mice were still able to find the three food rewards.

**Fig. 6:**
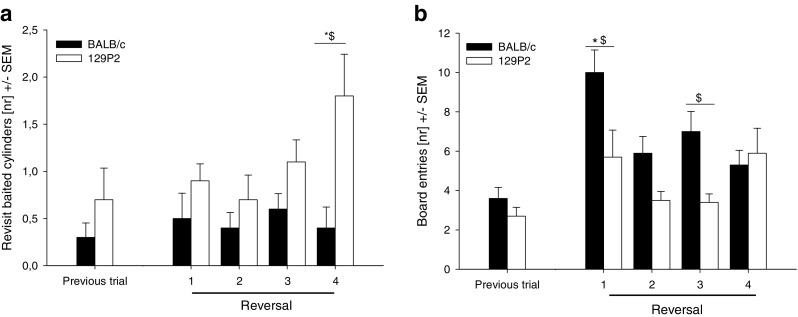
Cognitive parameters measured in BALB/c and 129P2 mice in the modified hole board during the reversal task. Data are shown as Trial 36 (previous trial) versus the reversal trials (Trials 37–40). (A) Number of revisits to baited cylinders, as an indicator for short-term memory errors. (B) Number of board entries. ^*^ *p* < .0085 (vs. previous trial). ^$^ *p* < .0085 (strain difference)

We observed a significant increase in the number of nonbaited cylinder visits (Fig. 5B) and wrong choices. Both strains showed more nonbaited cylinder visits and wrong choices in all four reversal trials than on the trial before the reversal (for BALB/c, *U* = −3.191, *p* < .0085, and *U* = −3.143, *p* < .0085, respectively; for 129P2, *U* = −4.082, *p* < .0085, and *U* = −2.756, *p* < .0085, respectively). The number of revisits to baited holes increased in the last reversal trial in 129P2 mice (*U* = −2.649, *p* < .0085), when compared to the trial before the reversal (Fig. 6A). No further effects on cognition-related parameters were found during the reversal task.

### Overall behaviour: Reversal task

Strain differences were found in the latencies until the first board entry; 129P2 mice showed longer latencies to enter the board in Reversal Trials 2, 3, and 4 than did the BALB/c mice. BALB/c mice showed an increased number of board entries (Fig. 6B) and line crossings in the first reversal trial when compared to the previous trial (*U*s = −3.604 and −3.759, respectively; *p*s < .0025), suggesting that the BALB/c mice increased their overall activity level during the first reversal trial, although no effects were found in the other three reversal trials. 129P2 mice showed an increased number of cylinder explorations in the last reversal trial as compared to the trial before the reversal (*U* = −2.276, *p* < .0025). No further effects on behaviour were found during the reversal trial. In Table 2, the behavioural data for the first trial, the trial prior to the reversal (Trial 36), and the first reversal trial (Trial 37) are summarized.

**Table 2:** Overview of behavioural parameters measured in the modified hole board

Behavioural Category				BALB/c			129P2	
	Behavioural Parameter	ANOVA Effects^@^	Trial 1	Trial 36	Trial 37 (First Reversal)	Trial 1	Trial 36	Trial 37 (First Reversal)
Cognition	Latency end task	T, S	300.0 ± 0.0	38.3 ± 6.6	90.0 ± 6.8	296.8 ± 3.2	68.5 ± 8.2	119.5 ± 23.1
	Latency first visit baited hole	T, S, T × S	238.2 ± 28.7	11.7 ± 4.5	29.1 ± 3.1	282.2 ± 12.0	16.1 ± 3.1	37.3 ± 10.9
	Omission errors	T, S, T × S	3 (0)	0 (0)	0 (0)	3 (0.5)	0 (0)	0 (0)
	Revisit baited hole	T, S, T × S	0 (0)	0 (0.5)	0 (0.5)	0 (0)	0 (1)	1 (0)
	Visit nonbaited hole	T, S, T × S	2 (1.5)	0 (0)	3 (4)	1 (0.5)	0 (0)	3 (2)
	Wrong choice	T, S, T × S	2 (2)	0 (0.5)	3 (2)	1 (0.5)	0 (1)	4 (2)
Avoidance	Latency board	T, T × S	31.8 ± 5.4	4.1 ± 4.0	6.5 ± 1.6	17.4 ± 1.7	12.4 ± 3.0	11.5 ± 1.9
	Time spent on board	T, T × S	81.9 ± 4.3	66.6 ± 5.6	55.2 ± 3.2	92.9 ± 0.8	55.9 ± 6.5	53.0 ± 5.4
	Board entries	T, S	5 (4.0)	3.5 (2.5)	10 (3)	2 (1.5)	2 (1)	5 (3)
Risk assessment	Stretched attends	T, T × S	42 (18)	0 (0)	0 (0)	27 (4.5)	0 (0)	0 (0)
	Latency stretched attend	T, S, T × S	3.6 ± 0.5	300.0 ± 0.0	300.0 ± 0.0	4.4 ± 0.9	300.0 ± 0.0	300.0 ± 0.0
Locomotion	Line crossings	T, S, T × S	2 (5)	12 (4.5)	28 (14)	2 (0.5)	5 (7.5)	13 (13)
	Latency line crossing	T, S	13.4 ± 4.4	1.5 ± 0.2	1.5 ± 0.1	98.2 ± 43.2	4.5 ± 1.3	1.8 ± 0.3
	Time spent immobile	S	0.0 ± 0.0	0.0 ± 0.0	0.0 ± 0.0	0.37 ± 0.37	0.0 ± 0.0	0.0 ± 0.0
	Latency immobility	T, S	300.0 ± 0.0	300.0 ± 0.0	300.0 ± 0.0	283.3 ± 16.7	300.0 ± 0.0	300.0 ± 0.0
Exploration	Rearings box	T, S	0 (1)	0 (1.5)	2 (2)	0 (0)	0(0)	1 (3)
	Rearings board	T	0 (1)	0 (0)	0 (0)	0 (0.5)	0 (0)	0 (0)
	Hole explorations	T, S, T × S	20 (18)	2 (4)	7 (3)	16 (12.5)	2 (2)	4 (4)
Arousal	Time spent grooming	S	0.48 ± 0.1	0.3 ± 0.3	0.5 ± 0.2	0.3 ± 0.2	0.2 ± 0.2	0.0 ± 0.0
	Latency grooming	S	255.3 ± 15.6	274.4 ± 25.6	230.5 ± 35.9	285.0 ± 10.0	279.6 ± 20.4	300.0 ± 0.0
	Defecations	T	4 (6)	0 (0)	0 (0)	3 (4)	0 (0)	0 (1)

Data for the 1st trial, the 36th trial, and the first reversal trial are shown as means ± *SEM*s for continuous data; ordinal data are shown as medians with an interquartile range in parentheses. ^@^ANOVA effects significant at *p* < .05: T = trial effect; S = strain effect; T × S = Trial × Strain interaction effect.

## Discussion

In general, both the 129P2 and the BALB/c mouse strains successfully acquired the mHB task and improved their cognitive performance over time (Fig. 2A). However, the 129P2 mice needed more time to learn to find all of the food rewards, as indicated by a higher number of omission errors during the first week of testing (Fig. 2B). During this same time period, BALB/c mice revealed an increase in the number of wrong choices (Fig. 3a). While recollection of the previously learned information is needed to avoid long-term memory errors (Squire & Zola, 1996), this increase in wrong choices may not reflect a cognitive impairment, as it was paralleled by a rapid decrease in omission errors. Together, these observations suggest that these mice simply shifted from a short, initial behavioural inhibition to active exploration of the mHB and its cylinders. Only later—that is, after the first week of testing—did both strains improve their long-term memory performance and reach stable low error rates of near zero. Similar findings have been observed in a comparable study by Ohl et al. (2003): The initially highly anxious DBA/2 strain revealed, after rapid habituation to the testing situation, good cognitive performance based on more directed exploration of the hole board. A comparable strategy was found here for BALB/c mice: A rapid decrease in anxiety-related behaviour and an increase in exploration seemed to result in successful cognitive performance.

In contrast, 129P2 mice have been characterised by delayed habituation to the testing environment (Salomons et al., 2010), and this delay in habituation in 129P2 mice was paralleled by a delayed learning curve when compared to the BALB/c animals (Figs. 3A and B). Nevertheless, 129P2 mice eventually reached the same level of long-term memory performance in the course of the testing procedure, proving that their ability to learn a visuospatial task was not impaired in general, but was the result of a habituation-related exploratory strategy. This finding is in contrast with the literature regarding general memory performance in 129 mouse strains: Although studies concerning memory performance in the 129P2 mouse strain are scarce, it has been reported that some 129 substrains show clear memory deficits in different cognitive tests, such as the Morris water escape task and habituation in the open field test (Bothe, Bolivar, Vedder, & Geistfeld, 2004, 2005; Brooks, Pask, Jones, & Dunnett, 2005; Montkowski, Poettig, Mederer, & Holsboer, 1997). However, these studies did not analyse habituation profiles in the substrains they tested, and it cannot be excluded that the animals’ cognitive performance in these studies was confounded with impairments in habituation, probably resulting in delayed, but not generally impaired, cognitive performance. Maybe even more important is that some of these specific substrains show great impairment in locomotor activity. The memory deficits observed in the open field and Morris water maze could thus be attributed to low exploratory and locomotor activity in general. However, results from this study and from a previous study (Salomons et al., 2010) have shown that the specific 129P mouse strains we tested show similar exploratory and locomotor activity, as compared to BALB/c mice, and are not impaired in their general performance in learning a visual–spatial task.

In contrast, short-term memory (Fig. 3B) in 129P2 animals did not stabilise in the course of the experiment, but their error rate remained at a higher level than in the BALB/c mice. In BALB/c mice, as in other mouse strains (Ohl et al., 2003; Ohl et al., 2002), the increase in short-term memory errors during the initial phase of testing can be attributed to the process of learning about the characteristics of the test—that is, learning which cylinders are baited and that only one food reward per cylinder can be retrieved. 129P2 mice, however, make significantly more of this type of memory error than do BALB/c animals, which is particularly evident after stable long-term memory performance has been reached. Notably, the number of short-term memory errors even increased during the reversal session in 129P2 mice, an effect that points towards a deficit in cognitive flexibility in the 129P2 strain (Fig. 4). It is known that the prefrontal cortex plays a crucial role in the mediation of short-term memory processes (Dalley, Cardinal, & Robbins, 2004; Goldman-Rakic, 1996; Ongur & Price, 2000), as well as in processing behavioural flexibility (Dalley et al., 2004) and in mediating shifts between new strategies and rules (Brown & Bowman, 2002; de Bruin, Sanchez-Santed, Heinsbroek, Donker, & Postmes, 1994; Ragozzino, 2007). Reversal learning in general is described as a relatively low short-order rule and is predominantly regulated by the orbitofrontal cortex, while switching strategies involves the medial prefrontal cortex. In our study, the reversal was not simply a change in contingency, but mice needed to learn that the previously marked cylinders were now nonbaited and that unmarked cylinders were baited—that is, they needed to learn about a change of the rules.

A potential role of the prefrontal cortex in the habituation profile of 129P3 mice has previously been suggested by our group. We reported lower c-Fos activity in the prefrontal cortex after repeated exposure to the mHB in 129P3 mice when compared to BALB/c animals (Salomons et al., 2010). Furthermore, impaired fear extinction was found across several 129 substrains, a process based on an intact and functional corticolimbic circuit (Camp, Norcross, Whittle, Feyder, D’Hanis, Yilmazer-Hanke, Singewald & Holmes, 2009). Together with the observed impairment in cognitive flexibility in 129P2 mice, these findings suggest that impaired cognitive flexibility and its neurobiological underpinnings may indeed explain both the behavioural and cognitive performance of these animals in the mHB test.

In summary, the present study shows that both BALB/c and 129P2 mice are able to learn a visuospatial learning task. The initially highly anxious BALB/c mice showed rapid habituation to the test setup, followed by a successful acquisition of the mHB task. By contrast, 129P2 mice showed an impaired habituation profile, confirming our previous findings. This profile appeared to be accompanied by a specific exploratory strategy, in line with human studies (Wilken, Smith, Tola, & Mann, 2000), but was not paralleled by a general cognitive impairment. However, impaired habituation in 129P2 animals was associated with a delay in learning the task, a deficit in cognitive flexibility, and worse short-term memory performance as compared to BALB/c mice. These memory deficits are known to be mediated by the prefrontal cortex and have been observed in patients suffering from anxiety disorders (Ferreri et al., 2011). Together, these results support our hypothesis and demonstrate that the nonadaptive anxiety phenotype in 129P2 mice may indeed involve impaired cognitive control of emotional processes, resulting in impaired behavioural flexibility, for example in response to novelty.

Nevertheless, the results described here are inconclusive as to whether or not cognitive (dys)function indeed precedes the nonadaptive anxiety profile that we observed in 129P2 mice. It may very well be that the memory performance of 129P2 mice described in this study is the result of their disability in habituating to the test situation. This relationship remains a challenging issue to investigate (and is not one to be solved easily). The 129P2 mouse strain seems a promising new model to further investigate this relationship between anxiety and cognition and its underlying mechanisms.
